# Structure Identification of Adsorbed Anionic–Nonionic Binary Surfactant Layers Based on Interfacial Shear Rheology Studies and Surface Tension Isotherms

**DOI:** 10.3390/molecules28052276

**Published:** 2023-02-28

**Authors:** Ourania Oikonomidou, Margaritis Kostoglou, Thodoris Karapantsios

**Affiliations:** Department of Chemical Technology and Industrial Chemistry, Faculty of Chemistry, Aristotle University of Thessaloniki, University Box 116, 541 24 Thessaloniki, Greece

**Keywords:** sodium oleate, surfactant binary mixtures, adsorption, synergism, interfacial shear rheology, viscoelasticity, surface tension isotherms

## Abstract

Mixtures of anionic sodium oleate (NaOl) and nonionic ethoxylated or alkoxylated surfactants improve the selective separation of magnesite particles from mineral ores during the process of flotation. Apart from triggering the hydrophobicity of magnesite particles, these surfactant molecules adsorb to the air–liquid interface of flotation bubbles, changing the interfacial properties and thus affecting the flotation efficiency. The structure of adsorbed surfactants layers at the air–liquid interface depends on the adsorption kinetics of each surfactant and the reformation of intermolecular forces upon mixing. Up to now, researchers use surface tension measurements to understand the nature of intermolecular interactions in such binary surfactant mixtures. Aiming to adapt better to the dynamic character of flotation, the present work explores the interfacial rheology of NaOl mixtures with different nonionic surfactants to study the interfacial arrangement and viscoelastic properties of adsorbed surfactants under the application of shear forces. Interfacial shear viscosity results reveal the tendency on nonionic molecules to displace NaOl molecules from the interface. The critical nonionic surfactant concentration needed to complete NaOl displacement at the interface depends on the length of its hydrophilic part and on the geometry of its hydrophobic chain. The above indications are supported by surface tension isotherms.

## 1. Introduction

Vulnerable multiphase systems such as foams and emulsions exist at the majority of chemical products, such as processed food, beverages, cleaning detergents, medicine and cosmetics [1,2,3]. The stability of these systems strongly depends on mass transport phenomena and chemical interactions ongoing at the region of the involving interfaces. Thus, understanding the interactions between bubbles and droplets, so as to control the quality of foam and emulsions in products, requires a deep understanding of the ongoing dynamic and equilibrium interfacial properties [3,4,5]. In this manner, a plethora of research works examine the interfacial properties of bubbles and droplets in the presence of surface active molecules (i.e., surfactants) [1,4,6,7,8,9,10,11]. The amphiphilic nature of these molecules makes them adsorb to the incorporating gas/liquid interfaces and prevent them from collapse [12]. The technical and economic advantages of using surface active molecules as mixtures and not as individuals have been reported several times in the literature. Proper surfactant combinations can achieve better stabilizing properties at much lower concentrations; therefore, in practice, binary surfactant mixtures are applied quite often in chemical processes [1,6,7,10]. Mixing molecules of different chemical structure introduces the complexity of competitive adsorption and intermolecular interactions to the resulting interfacial arrangement [4].

Interaction parameter ‘β’ can reveal the nature and magnitude of surfactant interactions at the interface of a binary solution resulting from the reformation of intermolecular forces (i.e., electrostatic and steric repulsive forces, ion-dipole and Van der Waals attraction forces) upon mixing. Negative β values indicate synergistic interactions between the mixed surfactants, as the repulsive forces weaken and attractive forces develop instead. On the contrary, positive β values correspond to antagonistic interactions between mixed surfactants, as in this case, the repulsive forces between two different surfactants are stronger compared to the self-repulsive forces between two identical surfactant molecules [6,7,8,9,10,11,12,13]. To calculate the interaction parameter of a system, surface tension isotherms of the binary surfactant solution and the solutions of individual surfactant components need to be employed. Up to now, the interactions between numerous ionic–ionic and ionic- nonionic surfactant combinations have been tested for the purpose of generating formulas of better surface activity [6,7,8,9,10,11,14]. However, surface tension results are restricted to equilibrium conditions and lack the ability to provide dynamic information on the interfacial rearrangement of surfactant molecules under the application of stresses, as encountered in the majority of chemical processes [3].

To understand the stability and mobility response of adsorbed molecular layers under external forces, researchers perform interfacial rheology tests. Interfacial rheology is studied under both dilatational and shear deformation, since both of them are present in processes involving such multiphase systems [15]. Dilatational deformation changes the area but not the shape of the tested interface and depends on the nature of the molecules adsorbed on the interface [16]. Interfacial dilatational rheology of systems is extensively examined in the literature [1,17,18,19]. Liggieri and Miller., 2010, resume some critical studies on the dilatational rheology of adsorbing surfactant layers at liquid interfaces, while Miller et al., 1996, report the different instrument designs available for these studies [20,21]. On the other hand, shear deformation changes the shape but not the area of the tested interface and depends on intermolecular interactions between the different species [16,22].

Interfacial shear rheology is less exploited; however, there are some critical works that use it to identify the structure of adsorbed mixed layers as a result of different adsorption kinetics and intermolecular forces [14,23]. The majority of systems tested under shear deformation are surfactant–protein mixtures [24,25,26]. Contrary to small surfactant molecules, large protein molecules impose some significant resistance towards motion of the interface and allow such kind of measurements. In practice, small surfactant molecules co-exist with biodegradable large protein molecules in many food- and medicine-related systems. Some indicative examples are as follows: Kragel et al., 2008, review the interfacial shear rheology behavior of various protein–surfactant mixtures used as stabilizers in foams and emulsions employing several experimental techniques [4]. As they report, surfactant molecules tend to displace the adsorbed protein molecules from the interface, turning the elastic proteins properties into viscous. The displacement is dictated by the nature of the surfactant and the protein/protein interactions. Dwyer et al., 2012, studied the interaction of designed small unstructured peptides with large-structured protein molecules at the interface of air/water systems [2]. Synergistic interaction is reported as the adsorption kinetics of the mixture is higher than that of individual molecules. Erni et al., 2003, present the results from steady shear and oscillatory experiments as well as creep recovery and stress relaxation tests for ovalbumin protein adsorbed films and sorbitan tristearate spread films on both oil/water and air/water interfaces [27]. Torcello-Gómez et al., 2011, studied the surface rheology of sorbitan tristearate (food emulsifier and stabilizer) and b-lactoglobulin (protein present in cow milk) mixtures with both shear and dilatational deformation tests [16]. Amongst the few surfactant–surfactant mixtures tested as a matter of interfacial rheology is sodium oleate—C12(EO)6, aiming to understand the effect of surfactants concentration on foam drainage during the process of flotation deinking [28]. However, in this case, the resulting surface properties are restricted as derived by dynamic surface tension measurements.

In the field of flotation, sodium oleate (NaOl) is a rather valuable surfactant as it is considered a very compatible collector for the recovery of magnesite particles from mineral ores [29,30]. The addition of nonionic cocollectors to the primary anionic NaOl is believed to enhance further the recovery rates [31]. The efficiency of flotation depends on the hydrodynamics at the interface of bubbles that may affect the motion of the bubble through the liquid [15,32]. It is worth noticing that shear interfacial viscosity has no effect on the motion of a bubble due to an external force field (e.g., gravity). This has been proved by solving the corresponding set of the fluid dynamics equations [33,34]. The axial symmetry of the resulting flow field prevents the influence of interfacial shear viscosity. However, in case of three-dimensional turbulent flow conditions met in flotation, there may be an effect of interfacial shear velocity on altering the flow field around the bubble. The interfacial shear viscosity is of paramount importance for froth drainage through Plateau borders [35,36]. Up to now, researchers study the collector–cocollector interactions through surface tension isotherms [37]. Going a step further, the present work attempts to examine the structure and viscoleasticity of adsorbing mixed surfactant layers at the air/liquid interface of flotation systems, by studying the interfacial rheology of different NaOl and ethoxylated/alkoxylated nonionic surfactant mixtures under the application of shear forces. Surface tension isotherms are performed as supporting indications of the adsorbing surfactant layers’ structure. The structure of this work is the following: the Materials and Methods section presents the employed surfactant reagents and experimental devices. Experimental measurements are presented and extensively discussed in the Results and Discussion section. Finally, the conclusions are presented in the last section of this work.

## 2. Results and Discussion

Table 1 reports the pH values of 1CMC NaOl solution and anionic-nonionic binary mixtures at the maximum tested nonionic concentration. Measurements show that all pH values are close to 10 since the addition of nonionic surfactants in 1CMC NaOl solution does not much affect the pH. At this level of pH, the flotation process is efficient, so no further pH regulation is needed to consider the following intermolecular results as appropriate for making conclusions [38].

**Figure 1:**
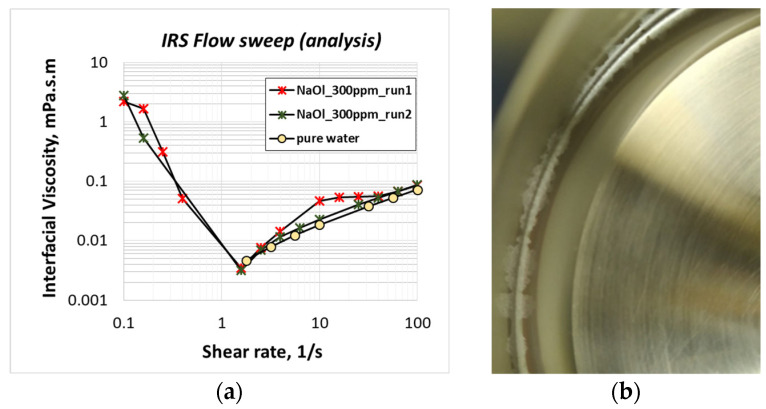
(**a**) Interfacial viscosity of undissolved sodium oleate (NaOl) particles precipitates under various shear rates, at 15 °C working temperature; (**b**) layer of NaOl solid particles precipitating at the solution surface before rotation of the bicone geometry.

Figure 1a shows the shear viscosity of anionic NaOl collector molecules adsorbing on the air/liquid interface of two different 1CMC (300 ppm) NaOl solutions at 15 °C. The results are quite repeatable. The artificial shear viscosity of a ‘surfactants-free’ ultrapure water surface is reported as a supporting control measurement. Pure water viscosity numbers indicate the lower measuring limits of the rheometer and have no physical meaning. This explains the missing measuring points below 1/s shear rate that are discarded by the rheometer as not trusted, as they correspond to extremely low viscosity values. Comparing the interfacial shear viscosity values of NaOl solutions to those of pure water, it can be seen that the adsorbing NaOl molecules attain some measurable viscosity at low shear rates, below 1/s. Higher shear rates seem to break the interfacial NaOl layer leaving the liquid surface free of surfactant molecules. This experimental conclusion is verified by visual inspection of the liquid surface before and after each run (continuous and accelerating bicone rotation). Figure 1b shows NaOl layers on the liquid surface, perimetrically to the walls of the test cell, before the application of shear stresses. After each experimental run, these layers are no longer detected. The formation of NaOl layers on the liquid surface is an indication that NaOl is not soluble to water at 15 °C. Therefore, these layers are considered as aggregates of non-dissolved NaOl solid particles that precipitate on the liquid surface. Experiments show that the present working temperature is not applicable to the studied flotation systems. Thereafter, all experiments are conducted at 30 °C.

**Figure 2:**
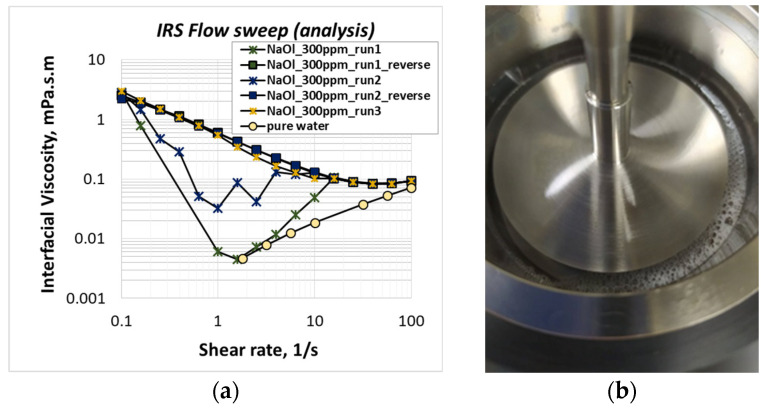
(**a**) Shear viscosity of NaOl molecules adsorbed at the interface of 1CMC NaOl solution at 30 °C; (**b**) NaOl solution foaming at the walls of the IRS test cell.

Figure 2a shows the shear viscosity of NaOl molecules adsorbed on the air/liquid interface for 1CMC (300 ppm) NaOl solution at 30 °C. At this temperature, NaOl is soluble to water. However, NaOl is a frother, and when the solution is poured into the test cell, it creates foam at the perimeter of the air/liquid interface (see Figure 2b). Runs 1 and 2 show the interfacial shear viscosity of two different 1CMC NaOl solutions. These measurements are not considered repeatable; however, they have a similar trend. Interfacial viscosity decreases with the increase in shear rate up to 1/s and has an unstable behavior at shear rates above 1/s. To investigate this behavior further, additional ‘reverse’ runs are performed by decreasing the shear rate from 100/s to 0.1/s with a logarithmic ramp. The measuring duration per shear rate does not change. The resulting reverse runs 1 and 2 are repeatable, indicating the following conclusions: under low shear rates (<10/s), the air trapped in the foam lowers the measured interfacial resistance, resulting in lower interfacial viscosity values than the real ones. Under high shear rates (>10/s), the fast bicone rotation breaks the foam, resulting in realistic viscosity measurements. In the case of reverse measurements that start under high shear rates, the foam breaks at the beginning of each run and allows us to attain proper viscosity measurements during the whole run. To overcome this problem, NaOl solution is poured slowly into the test cell to avoid foaming and manage proper (repeatable) interfacial viscosity measurements, as shown in run 3. Discussing on the measurement itself, it seems that the anionic NaOl molecules adsorbing on the air/liquid surface acquire some interfacial shear viscosity (all measuring values are above those of pure water). Furthermore, the adsorbed NaOl layer has a non-Newtonian shear thinning behavior, as its viscosity decreases with shear rate.

**Figure 3:**
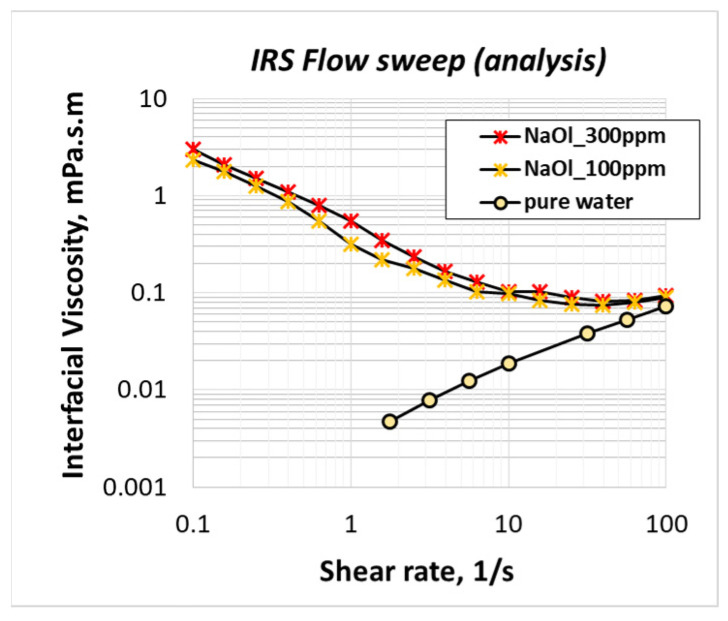
The effect of NaOl solution concentration at the interfacial viscosity of adsorbed NaOl molecules under various shear rates.

Figure 3 shows the variation of NaOl solution interfacial viscosity at the region of premicellar concentrations. As expected, the interfacial viscosity decreases with the decrease in NaOl concertation; however, the qualitative behavior of interfacial viscosity with shear rate remains the same. This indication is critical as in flotation applications the concentration of the collector is in the order of 100 ppm. Therefore, it seems that the interfacial rheology findings at 1CMC NaOl can also stand for the real flotation concentrations.

**Figure 4:**
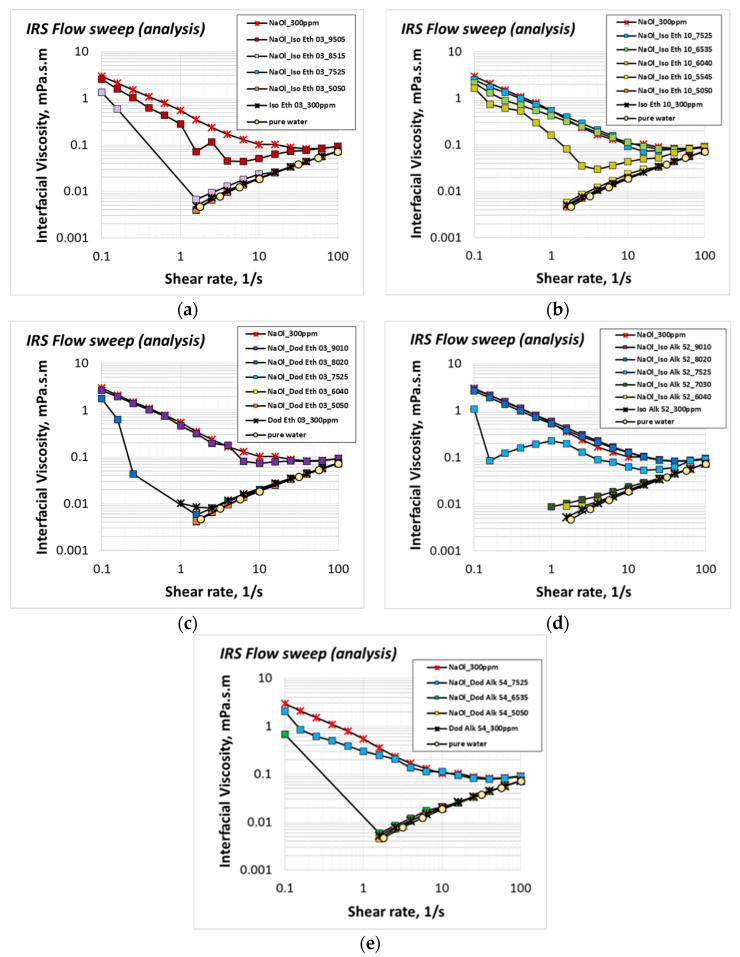
Shear viscosity of adsorbed surfactants layers at the interface of binary solutions of 1CMC NaOl collector and (**a**) Iso Eth 03; (**b**) Iso Eth 10; (**c**) Dod Eth 03; (**d**) Iso Alk 52; (**e**) Dod Alk 54; nonionic cocollector, at 30 °C.

**Figure 5:**
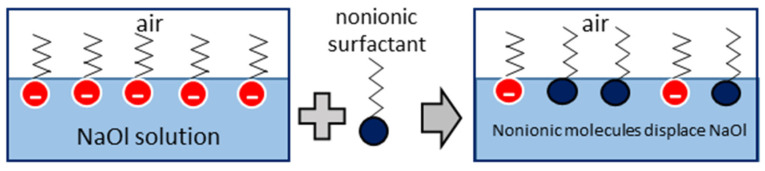
Nonionic cocollector molecules displace ionic primary collector molecules from the air/liquid interface of the binary solution.

In case flotation liquids contain binary collector mixtures, both anionic and nonionic surfactant molecules adsorb on the air–liquid interface, forming arrays based on the ongoing intermolecular forces. The structure of adsorbing binary surfactant monolayers is examined through interfacial rheology studies. Different cocollectors are tested at different anionic:nonionic mass ratios varying from 95:05 to 50:50. NaOl concentration at the test solutions is always 1CMC (300 ppm). Interfacial shear viscosity results are presented in Figure 4, accompanied by the corresponding control measurements of 1CMC NaOl, nonionic cocollector solution at the same concentration and pure water. The interfacial shear viscosity of nonionic cocollector solutions is insignificant and equal to that of pure water. This means that the adsorbing nonionic molecular layers do not impose any extra interfacial viscosity under shear stress. The gradual addition of a nonionic cocollector at 1CMC NaOl solution decreases the resulting shear viscosity of the surfactants binary layer adsorbed at the air/liquid interface. Above some critical nonionic mass ratio, the interfacial shear viscosity is reduced to that of the nonionic cocollector molecules. This trend exists for all cocollector cases, and it is a strong indication that by increasing the cocollector concentration in the binary solution, the cocollector molecules displace the adsorbed anionic NaOl collector molecules at the air/liquid interface. The proposed displacement mechanism is illustrated in Figure 5. Above the critical mass ratio, the air/liquid interface is occupied to the utmost by the nonionic surfactant molecules. The above observation is quite similar to that of Kragel et al., 2008, and Bosa and van Vlieta, 2001, reporting that the displacement of large protein molecules by surfactant molecules of low molecular weight changes the shear viscoelasticity of the adsorbed layer [4,39].

The critical nonionic cocollector mass ratio varies with the molecular structure of the surfactant. Table 2 summarizes the critical mass ratios of all tested nonionic cocollectors. Critical mass ratio increases for nonionic molecules with longer hydrophilic heads, either due to more ethoxylated groups (compare values of Isotridecyl Ethoxylate 03 and Isotridecyl Ethoxylate 10) or due to alkοxylated chains (compare values of Isotridecyl Ethoxylate 03 and Isotridecyl Alkoxylate 52, or values of Dodecyl Ethoxylate 03 and Dodecyl Alkoxylate 54). Molecules with an enhanced hydrophilic part are more soluble in the liquid phase, and thus, a greater concentration of these molecules is needed to cover the air/liquid interface. On the other hand, the critical mass ratio is lower for nonionic molecules with a branched hydrophobic chain (compare values of Isotridecyl Ethoxylate 03 and Dodecyl Ethoxylate 03). The Isotridecyl branched chain occupies larger space on the interface due to the strong stearic repulsive forces between the hydrophobic heads of nonionic molecules, and thus, fewer molecules are needed to cover the entire interface [8].

**Figure 6:**
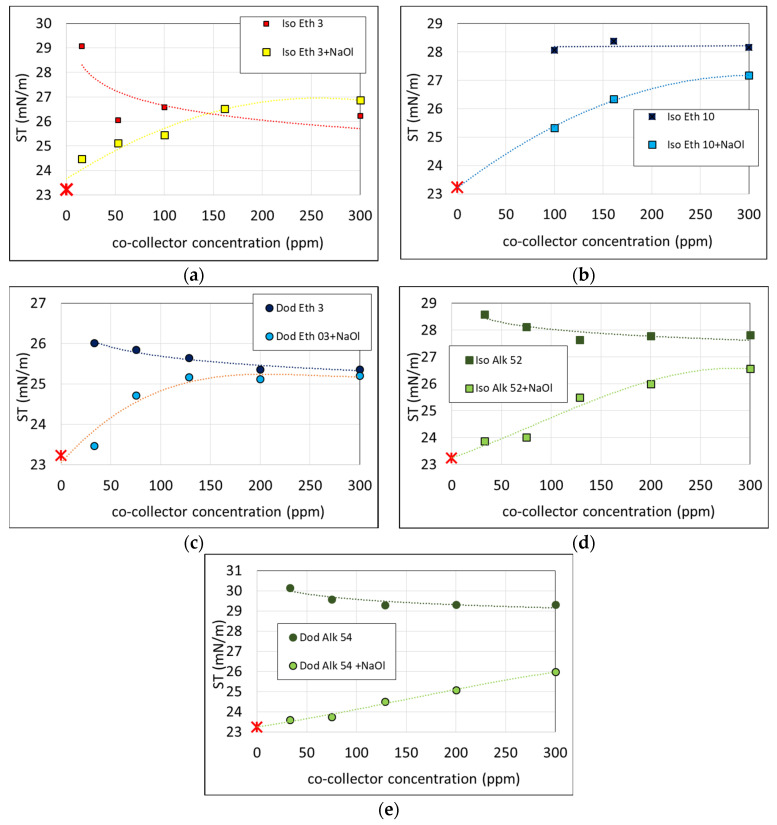
Surface tension (ST) isotherms of nonionic surfactant solutions: (**a**) Iso Eth 03; (**b**) Iso Eth 10; (**c**) Dod Eth 03; (**d**) Iso Alk 52; (**e**) Dod Alk 54; and anionic–nonionic binary mixtures at 30 °C, for 300 ppm NaOl (1CMC) and different nonionic surfactant concentrations at 30 °C. The red star symbol shows the surface tension of 1CMC NaOl solution.

Additional conventional surface tension isotherms at 30 °C verify the interfacial structure of flotation bubbles as resulting from the present rheological study. The results are presented in Figure 6. Increasing the concentration of each nonionic cocollector in water decreases the surface tension of the solution (dark-colored points of each subfigure). Surface tension becomes stable at 1CMC condition of each cocollector. Surface tension of the corresponding anionic–nonionic binary mixture increases by increasing the nonionic cocollector concentration in the solution (light-colored points of each subfigure). Surface tension at zero nonionic concentration corresponds to surface tension of 1CMC NaOl solution (marked with a red star). Measurements show that the CMC of all tested nonionic surfactant solutions is below CMC of NaOl (300 ppm). Thus, all tested nonionic cocollectors are more surface active than NaOl and dominate on the solution surface. This explains the fact that the surface tension of binary mixtures does not decrease with the increase in surfactants concentration, but it increases towards surface tension at 1CMC of nonionic surfactant solutions. The above conclusion agrees with the rheological finding that nonionic cocollector molecules displace NaOl molecules from the air/liquid interface. The nonionic cocollector concentration that makes surface tension of the binary solution equal to that of 1CMC nonionic surfactant solution, is the concentration needed to displace all NaOl molecules from the interface. This concentration is always higher than the corresponding critical mass ratio deriving from the interfacial viscosity measurements (Table 3 shows the nonionic surfactant concentration that corresponds to each mass ratio of the binary mixture). This means that even in the presence of some NaOl molecules on the interface, the shear viscosity of the adsorbed binary layer is insignificant. For the cases of Isotridecyl Ethoxylate 10, Isotridecyl Alkoxylate 52 and Dodecyl Alkoxylate 54, the maximum tested concentration (300 ppm) is not adequate for the full displacement of NaOl from the interface, since the surface tension of the binary mixture never reaches surface tension at 1CMC of each nonionic surfactant. The aforementioned observation is in line with the molecular structure of the surfactants. The long hydrophilic head of these surfactants makes them water soluble and increases the concentration needed to compete NaOl molecules on surface adsorption. This phenomenon is even stronger for the case of Dodecyl Alkoxylate 54 as its straight Dodecyl chain is less space demanding due to the weak steric repulsive forces between the neighboring hydrophobic heads.

Based on the literature, the synergistic efficiency between ionic and nonionic molecules is defined as follows: the total concentration of the mixed surfactant required to reduce the surface tension of the solvent to a given value is less than that of either individual surfactants. This condition refers to the concentrations of surfactants that adsorb to the interface of solvents and not to surfactants concentrations in the solutions bulk. In the literature, all works that result in synergistic ionic–nonionic interactions report the exact same trend of the surface tension isotherms variation; the surface tension isotherm of the binary mixture is always between those of the individual surfactants and very close to the nonionic one [6,9,11].

**Figure 7:**
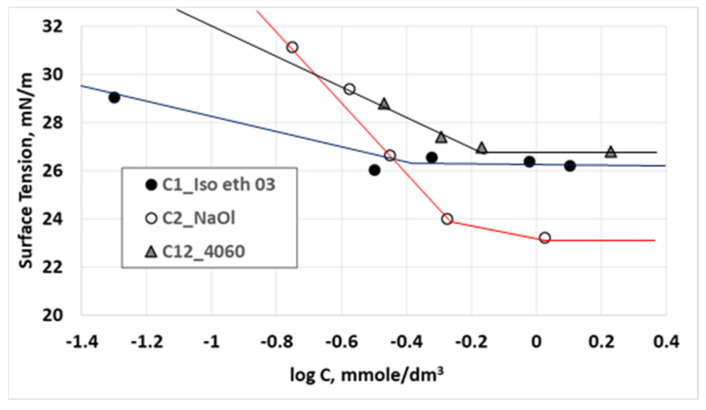
Surface tension variation with collectors concentration for NaOl solution, Iso Eth 03 solution and their binary mixture at 60:40 mass ratio.

Surface tension isotherms of Figure 7 aim to examine qualitatively the nature of intermolecular interactions at the indicative binary mixture of NaOl- Isotridecyl Ethoxylate 03. More specifically, Figure 7 presents the surface tension variation of Isotridecyl Ethoxylate 03 solution (collector 1), NaOl solution (collector 2) and their binary mixture (12), with the total collectors concentration. The mass ratio of the binary mixture is 40:60 (as at this mass ratio and for 1CMC NaOl concentration, the surface tension of the solution reaches surface tension of 1CMC Isotridecyl Ethoxylate 03 solution as shown in Figure 6a). Solid lines connect the surface tension measuring points to show the overall trend. Points at which trend lines become horizontal correspond to 1CMC values of the solutions. Expanding the surface tension trend lines of Figure 7 towards lower surfactant concentrations, shows that the present system has the same behavior with those reported in the literature [6,9,11]; the trend line of the mixture (in black) is between those of the individual surfactants (in blue and red). This finding indicates that for low-surfactant concentrations that correspond to surface tension values above 30 mN/m, NaOl interacts synergistically with Isotridecyl Ethoxylate 03. Calculation of interaction parameter β is not feasible for the present case, as the tested concentrations are above the pre-micellar regions of surfactant solutions. For the purpose of flotation research, it is meaningless to focus on lower surfactant concentrations; thus, the interaction between ionic and nonionic surfactants is only qualitatively estimated. In general, Zhou and Rosen., 2003, claim that repulsive (antagonistic) interactions are found only in mixtures of hydrocarbon chain and fluorocarbon chain surfactants of the same sign [8]. However, they straighten that interaction between an anionic surfactant and a nonionic surfactant with a polyoxyethylene chain of many oxyethylene units, acquires a weak positive charge that indicates a weak repulsive interaction nature [8,40,41,42]. NaOl—Isotridecyl Ethoxylate 10 system is an example with an expected repulsive behavior. Moreover, nonionic surfactants with branched hydrophobic chains affect the ionic–nonionic interactions, resulting in lower values of β parameter (stronger synergism); however, this effect considers mostly surfactants micellization in the bulk and not the surfactants interaction at the interface [10,12].

As a next step, shear viscoelasticity studies indicate the mobility and stability of surfactant layers adsorbed on the surface of flotation bubbles. The viscoelasticity of each binary system is tested at the critical mass ratio that results from the interfacial shear viscosity measurements. The reason is quite simple. Below critical mass ratios, the viscoelasticity of binary layers will be equal to that of individual NaOl molecules, and above the critical mass ratios, their viscoelasticity will resemble that of individual nonionic cocollector or pure water. The above conclusions derive from the interfacial shear viscosity measurements of Figure 4. Therefore, shear viscoelasticity of binary mixtures with a mass ratio diverging from the critical ones, are expected to coincide with one of the two aforementioned extreme tested conditions.

**Figure 8:**
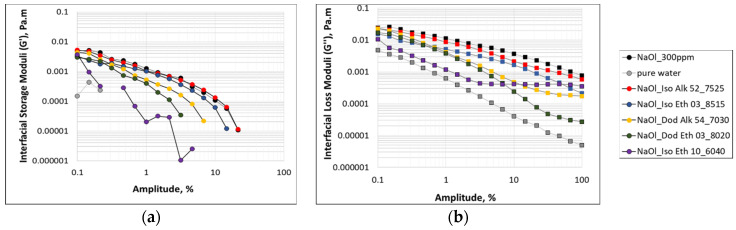
Oscillatory amplitude sweep runs under 1 rad/s constant frequency, to measure: (**a**) Interfacial storage modulus, G′, and (**b**) Interfacial loss modulus, G″, of 1CMC NaOl solution and NaOl-nonionic collector mixtures at their critical mass ratios.

At first, Figure 8 presents the amplitude sweep measurements that are performed to identify the linear viscoelastic region of the adsorbed binary surfactant layers. Amplitude sweep runs of 1CMC NaOl solution and pure water are reported as control measurements. The resulting storage modulus (G′) and loss modulus (G″) are presented in separate subfigures (Figure 8a,b in respect) so as to avoid data overcrowding. For the ease of comparison, the two subfigures have the same scaling. The results show that both storage and loss moduli of all binary mixtures at critical mass ratios are in between the corresponding moduli of 1CMC NaOl solution and the artificial measurements of pure water, meaning that the addition of nonionic cocollectors in the NaOl solution suppresses the magnitude of both elastic and viscous nature of the initial layer. This behavior is in consistency with the interfacial shear viscosity measurements of Figure 4. Moreover, the results show that for all tested binary mixtures and the anionic NaOl solution, and for the whole range of tested amplitudes, the loss modulus is approximately one order of magnitude higher than storage modulus. Since there is no intersection of the corresponding storage and loss moduli curves, the yield point cannot be identified. This clear domination of loss modulus elucidates the pure viscous behavior of all adsorbed surfactant layers, meaning that upon the application of some stress, the layers do not retain any memory of their initial condition. However, the absence of yield point shows that the structure of these layers does not break even under intense shear deformation. Another crucial point is that in both moduli measurements, it is not easy to detect the linear viscoelastic region of surfactant layers. The linear viscoelastic region is a very short part of the tested amplitude: 0.1–0.3%. The measuring sensitivity of the rheometer does not allow us to perform tests at lower amplitude values and obtain more clearly the linear viscoelastic region of the surfactant layers.

**Figure 9:**
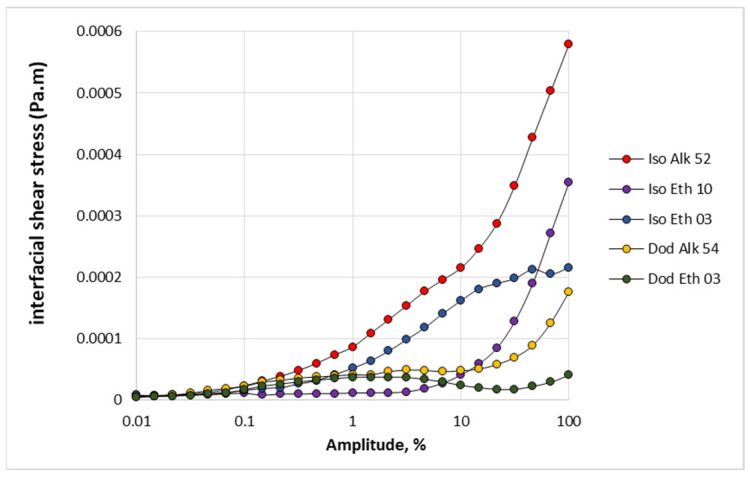
Interfacial shear stress applied on the interface of ionic–nonionic collector solutions under oscillations of different amplitude.

The interfacial shear stresses applied to the binary layers during amplitude sweep oscillations are reported in Figure 9. Deviations between the applied shear stresses become clear at amplitudes above 10%. Measuring data show that shear stress depends on the molecular structure of nonionic cocollectors. The most important factor that results in high shear stresses is the branched hydrophobic chain of cocollector. Comparing separately the shear stresses of brunched and straight cocollectors, it appears that the length of their hydrophilic chain is the second factor that results in high shear stress values [17].

**Figure 10:**
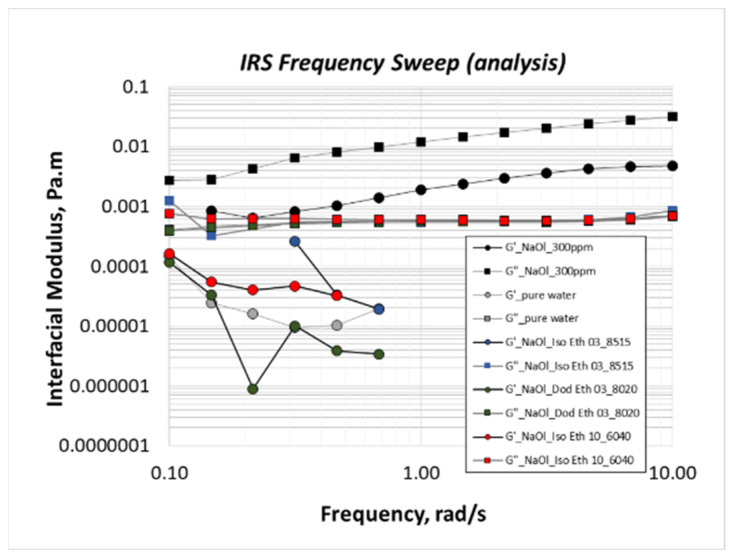
Oscillatory frequency sweep runs under 1% amplitude to measure the interfacial storage (G′) and loss (G″) moduli of 1CMC NaOl solution and NaOl-nonionic collector mixtures at their critical mass ratios.

Oscillatory frequency sweep runs should be performed for an amplitude in the linear viscoelastic region. For such low amplitudes, the resulting disturbance is not adequate to provide trustful measurements. Thus, the tested amplitude condition is a bit higher: 1%. Figure 10 shows the interfacial shear moduli of binary collector mixtures at the critical mass ratios. Measurements of 1CMC NaOl solution and pure water are reported as control conditions. The results show that both storage and loss moduli of binary mixtures coincide with those of pure water, indicating that the structure of layers break since the beginning of rotation. For oscillation conditions beyond the linear viscoelastic region, such a result is expected. Commenting on NaOl curves, the viscous character prevails along the whole frequency range, denoting the fluidic interface of flotation bubbles in the anionic collector solution.

## 3. Materials and Methods

**Figure 11:**
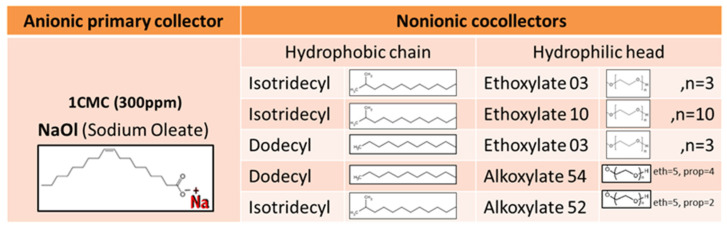
Molecular structure of anionic and nonionic surfactants that are tested as flotation collector and cocollectors in this work.

Sodium Oleate (Mw = 304.44 g/mol, ≥82%) is supplied from Sigma-Aldrich company. BASF company provided Isotridecyl Ethoxylate 03, Isotridecyl Ethoxylate 10, Dodecyl Ethoxylate 03, Dodecyl Alkoxylate 54 and Isotridecyl Alkoxylate 52 nonionic surfactants to be tested as cocollectors for flotation. For simplicity, from now on, the aforementioned nonionic cocollectors are named using the following abbreviations: Iso Eth 03, Iso Eth 10, Dod Eth 03, Dod Alk 54 and Iso Alk 52, respectively. These cocollectors are either water soluble, miscible or non-soluble in water. The chemical structure of all primary and cocollectors used in this work is illustrated in Figure 11.

A Millipore Direct-Q 3 UV water purification system is used to produce ultrapure water (Type 1) as a solvent for the preparation of test solutions. NaOl concentration in all tested surfactant solutions is 300 ppm (1CMC), as this concentration ensures the maximum accumulation of surfactant molecules on the solution surface without the formation of micelles in the bulk. The tested nonionic cocollector concentrations are calculated as mass ratios to the primary NaOl collector, ranging from 5:95 to 50:50. The dissolution of surfactants in water is performed under mild heating at 30 °C and mixing on a hot plate magnetic stirrer for 10 min. Additional ultrasonic mixing for 20 min helps to achieve the homogeneous dispersion of non-water-soluble surfactants in water bulk. Characterization of the resulting flotation liquids is performed at two working temperatures, 15 °C and 30 °C, that simulate ambient conditions during winter and summer months. The PH is a parameter that affects the intermolecular interactions between the different surfactants of a solution [43,44,45]. The pH of test solutions is measured using a HANNA INSTRUMENTS HI2020-edge Multiparameter pH meter equipped with a HI-10430 glass probe. 

The interactions between NaOl and nonionic cocollectors on the surface of flotation liquids indicate the structure of surfactant layers at the interface of the liquid and flotation bubbles. These interactions are examined through interfacial rheology and surface tension isotherms. Physica MCR 301 rheometer (Anton Paar) equipped with an Interfacial Rheology System (IRS) and a bicone geometry is used to study viscoelasticity at the interface between the test liquid solution and air under shear deformation. The diameter of the bicone is 60 mm, the diameter of the cylindrical test cell is 80 mm and the volume of the bottom phase (test solution) within the test cell is 110 mL. The test cell is covered with an evaporation trap to avoid disturbance of the examined interface. A Peltier element is placed at the bottom of the test cell to regulate temperature of the test solution at the desired level. Continuous air supply and Fisher Scientific Isotemp 3013 circulating chiller water bath are used to cool down the Peltier element. The application of a Controlled Shear Rate (CSR) measuring mode gives the interfacial viscosity of surfactant solutions in a wide range of shear rates (0.1–100/s). Five measuring points are recorded per decade of shear rate (logarithmic scale). The measuring duration of each point decreases from 8 min to 0.5 min with a logarithmic scale ramp while moving from low to high shear rate values. In case of low shear deformation, the measuring points are not considered trusted, and the rheometer does not transform the bulk data to interfacial data. Therefore, in some cases, the number of measuring points per decade of shear deformation can be less than five. Oscillatory motion is applied to study the interfacial viscoelasticity of surfactant solutions. The interfacial storage (G′) and loss (G″) moduli measurements resulting from amplitude sweep runs show the linear viscoelastic region of surfactants layer adsorbing on the surface of test solutions. During these measurements, bicone geometry oscillates with 1 rad/s constant frequency and amplitude escalating from 0.1 to 100% with a logarithmic time ramp from 3 min to 30 s. The applied frequency is low in order to avoid violent disturbances that would immediately break the thin interfacial layer structure. Oscillatory frequency sweep runs give the interfacial storage and loss moduli under a constant amplitude in the range of the resulting linear viscoelastic region and a frequency increasing from 0.1 rad/s to 10 rad/s, with a logarithmic time ramp from 3 min to 0.5 min. Both interfacial viscosity and interfacial viscoelastic moduli values result from a numerical hydrodynamic analysis on the flow field of the bulk phase [27]. 

A LAUDA TE2 tensiometer is used to measure the surface tension of test liquids with Wilhelmy plate method. The tensiometer is connected to a Brookfield TC-102 water bath for thermalization of test liquids at the desired working temperature. Upon determination of liquid surface location, surface tension measurements are recorded every 1 s.

## 4. Conclusions

The present work studies the individual and interactive performance of sodium oleate (NaOl) and nonionic collectors on the air–water interface related to flotation system reagents. Rheological and surface tension measurements illustrate the structure and viscoelasticity of interfacially adsorbed binary surfactant layers. Interfacial shear viscosity measurements under controlled shear rate indicate interactions between different types of adsorbed surfactant molecules on air–liquid interface. The adsorption of nonionic cocollectors on air–water interface does not change the interfacial viscosity of pure water surface. On the other hand, the adsorption of NaOl anionic collector molecules on the surface of 1CMC aqueous solution gives a significant interfacial viscosity that decreases with the applied shear rate. To obtain reliable results, the working temperature of the liquid must be above the room temperature so that NaOl is fully dissolved in water. The interfacial shear viscosity of anionic–nonionic binary collector mixtures shows that NaOl molecules adsorbed on the bubble surface are gradually displaced by nonionic cocollector molecules. NaOl molecules displacement is completed at lower anionic:nonionic *w*/*w* mass ratios for nonionic molecules of long hydrophilic head and branched hydrophobic chain. Surface tension isotherms show the synergistic interaction between NaOl and the tested nonionic cocollector molecules. Interfacial shear oscillatory runs show the pure viscous behavior of all binary surfactant layers under the whole range of tested amplitudes. The emerging linear viscoelastic region is quite short. The implementation of interfacial dilatational rheology studies in future will complete the examination of dynamic surfactant interactions for the present systems.

## Figures and Tables

**Table 1:** Measured pH of 1CMC NaOl solution and ionic–nonionic surfactant mixtures at 50:50 mass ratio.

Solution	pH
300 ppmNaOl	9.8
300 ppmNaOl + 300 ppm Iso Eth 10	9.6
300 ppmNaOl + 300 ppm Iso Eth 03	9.8
300 ppmNaOl + 300 ppm Dod Eth 03	9.8
300 ppmNaOl + 300 ppm Dod Alk 54	9.2
300 ppmNaOl + 300 ppm Iso Alk 52	9.2

**Table 2:** Regions of critical noninonic cocollector mass ratios resulting from the interfacial shear viscosity measurements of the tested ionic–nonionic binary mixtures.

Binary System	Critical Nonionic Concentration
NaOl—Iso Eth 03	15–25%
NaOl—Iso Eth 10	40–45%
NaOl—Dod Eth 03	20–25%
NaOl—Dod Alk 54	40–50%
NaOl—Iso Alk 52	25–30%

**Table 3:** The nonionic cocollector concentration corresponding to each anionic:nonionic binary collectors solution mass ratio. Anionic concentration is 300 ppm for all cases.

Nonionic Cocollector Concentration (ppm)	Anionic:Nonionic Binary Mixture Mass Ratio
15.7	95:05
33.3	90:10
52.9	85:15
75	80:20
100	75:25
128.5	70:30
161.5	65:35
200	60:40
270	55:45
300	50:50

## Data Availability

Data are available on request, due to restrictions.

## References

[1] Aono K., Suzuki F., Yomogida Y., Hasumi M., Kado S., Nakahara Y., Yajima S. (2020). Effects of Polypropylene Glycol at Very Low Concentrations on Rheological Properties at the Air−Water Interface and Foam Stability of Sodium Bis(2-ethylhexyl)sulfosuccinate Aqueous Solutions. Langmuir.

[2] Dwyer M.D., He L., James M., Nelson A., Middelberg A.P.J. (2012). Insights into the role of protein molecule size and structure on interfacial properties using designed sequences. J. R. Soc..

[3] Erni P., Fischer P., Windhab E.J., Fischer P., Marti I., Windhab E.J. Rheology of surfactant assemblies at the air/liquid and liquid/liquid interface. Proceedings of the 3rd International Symposium on Food Rheology and Structure, Zürich, Switzerland, 9–13 February 2003.

[4] Krägel J., Derkatch S.R., Miller R. (2008). Interfacial shear rheology of protein–surfactant layers. Adv. Colloid Interface Sci..

[5] Ferrari M., Navarini L., Liggieri L., Ravera F., Liverani F.S. (2007). Interfacial properties of coffee-based beverages. Food Hydrocoll..

[6] Bagheri A., Khalili P. (2017). Synergism between non-ionic and cationic surfactants in a concentration range of mixed monolayers at an air–water interface. RSC Adv..

[7] Zawala J., Wiertel-Pochopien A., Kowalczuk P.B. (2020). Critical Synergistic Concentration of Binary Surfactant Mixtures. Minerals.

[8] Zhou Q., Rosen M.J. (2003). Molecular Interactions of Surfactants in Mixed Monolayers at the Air/Aqueous Solution Interface and in Mixed Micelles in Aqueous Media: The Regular Solution Approach. Langmuir.

[9] El-Aila H.J.Y. (2009). Interaction of Nonionic Surfactant Triton-X-100 with Ionic Surfactants. J. Dispers. Sci. Technol..

[10] Rosen M.J., Zhou Q. (2001). Surfactant-Surfactant Interactions in Mixed Monolayer and Mixed Micelle Formation. Langmuir.

[11] Sis H., Chander G., Chander S. (2005). Synergism in Sodium Oleate/Ethoxylated Nonylphenol Mixtures. J. Dispers. Sci. Technol..

[12] Bagheri A., Abolhasani A. (2015). Binary mixtures of cationic surfactants with triton X-100 and the studies of physicochemical parameters of the mixed micelles. Korean J. Chem. Eng..

[13] Bosa M.A., van Vlieta T. (2001). Interfacial rheological properties of adsorbed protein layers and surfactants: A review. Adv. Colloid Interface Sci..

[14] Franck A. (2005). TA Instruments Germany. Interfacial Rheometry and the Stability of Foams and Emulsions.

[15] Edwards D.A., Brenner H., Wasan D.T. (2013). Interfacial Transport Processes and Rheology.

[16] Torcello-Gómez A., Maldonado-Valderrama J., Gálvez-Ruiz M.J., Martín-Rodríguez A., Cabrerizo-Vílchez M.A., de Vicente J. (2011). Surface rheology of sorbitan tristearate and b-lactoglobulin: Shear and dilatational behavior. J. Nonnewton Fluid. Mech..

[17] Erni P., Fischer P., Windhab E.J. (2005). Sorbitan Tristearate Layers at the Air/Water Interface Studied by Shear and Dilatational Interfacial Rheology. Langmuir.

[18] Ravera F., Loglio G., Kovalchuk V.I. (2010). Interfacial dilational rheology by oscillating bubble/drop methods. Curr. Opin. Colloid Interface Sci..

[19] Moradi N., Zakrevskyy Y., Javadi A., Aksenenko E.V., Fainerman V.B., Lomadze N., Santer S., Miller R. (2016). Surface tension and dilation rheology of DNA solutions in mixtures with azobenzene-containing cationic surfactant. Colloids Surf. A Physicochem. Eng. Asp..

[20] Liggieri L., Miller R. (2010). Relaxation of surfactants adsorption layers at liquid interfaces. Curr. Opin. Colloid Interface Sci..

[21] Miller R., Wüstneck R., Krägel J., Kretzschmar G. (1996). Dilational and shear rheology of adsorption layers at liquid interfaces. Colloids Surf. A Physicochem. Eng. Asp..

[22] Krägel J., Clark D., Wilde P., Krägel J., Miller R., Appell J., Porte G. (1995). Studies of adsorption and surface shear rheology of mixed β-lactoglobulin/surfactant systems. Trends in Colloid and Interface Science IX. Progress in Colloid & Polymer Science.

[23] Ruhs P.A., Boni L., Fuller G.G., Inglis R.F., Fischer P. (2013). In-Situ Quantification of the Interfacial Rheological Response of Bacterial Biofilms to Environmental Stimuli. PLoS ONE.

[24] Kragel J., Siegel S., Miller R., Born M., Schano K.H. (1994). Measurement of interfacial shear rheological properties: An automated apparatus. Colloids Surf. A Physicochem. Eng. Asp..

[25] Kotsmar C., Pradines V., Alahverdjieva V.S., Aksenenko E.V., Fainerman V.B., Kovalchuk V.I., Krägel J., Leser M.E., Miller R. (2009). Thermodynamics, adsorption kinetics and rheology of mixed protein-surfactant interfacial layers. Adv. Colloid Interface Sci..

[26] Dan A., Gochev G., Kragel J., Aksenenko E.V., Fainerman V.B., Miller R. (2013). Interfacial rheology of mixed layers of food proteins and surfactant. Curr. Opin. Colloid Interface Sci..

[27] Erni P., Fischer P., Windhab E.J. (2003). Stress- and strain-controlled measurements of interfacial shear viscosity and viscoelasticity at liquid/liquid and gas/liquid interfaces. Rev. Sci. Instrum..

[28] Beneventi D., Pugh R.J., Carré B., Gandini A. (2003). Surface rheology and foaming properties of sodium oleate and C12(EO)6 aqueous solutions. J. Colloid Interface Sci..

[29] Basarová P., Bartovská L., Korínek K., Horn D. (2005). The influence of flotation agent concentration on the wettability and flotability of polystyrene. J. Colloid Interface Sci..

[30] Zhang H., Liu W., Han C., Hao H. (2018). Effects of monohydric alcohols on the flotation of magnesite and dolomite by sodium oleate. J. Mol. Liq..

[31] Sis H., Chander S. (2003). Improving froth characteristics and flotation recovery of phosphate ores with nonionic surfactants. Min. Eng..

[32] Brabcová Z., Karapantsios T., Kostoglou M., Basarová P., Matis K. (2015). Bubble–particle collision interaction in flotation systems. Colloids Surf. A Physicochem Eng. Asp..

[33] Narsimhan V. (2018). Letter: The effect of surface viscosity on the translational speed of droplets. Phys. Fluids.

[34] Levan M.D. (1981). Motion of a Droplet with a Newtonian Interface. J. Colloid Interface Sci..

[35] Langevin D. (2000). Influence of interfacial rheology on foams and emulsion properties. Adv. Colloid Interface Sci..

[36] Nguyen A.V., Schulze H.J. (2004). Colloidal Science of Flotation.

[37] Chen C., Zhu H., Sun W., Hu Y., Qin W., Liu R. (2017). Synergetic Effect of the Mixed Anionic/Non-Ionic Collectors in Low Temperature Flotation of Scheelite. Minerals.

[38] Wonyen D.G., Kromah V., Gibson B., Nah S., Chelgani S.C. (2018). A Review of Flotation Separation of Mg Carbonates (Dolomite and Magnesite). Minerals.

[39] Rosen M.J., Yuan Hua X. (1982). Surface concentrations and molecular interactions in binary mixtures of surfactants. J. Colloid Interface Sci..

[40] Rosen M.J., Zhao F.J. (1983). Binary mixtures of surfactants. The effect of structural and microenvironmental factors on molecular interaction at the aqueous solution/air interface. J. Colloid Interface Sci..

[41] Nagarajan R. (2001). New Horizons: Detergents for the New Millennium Conference Invited Papers.

[42] Nagarajan R., Kalpakci B. (1982). Visometric investigation of complexes between polyethyleneoxide and surfactant micelles. Polym. Prepr. Am. Chem. Soc..

[43] Żamojć K., Wyrzykowski D., Chmurzyński L. (2022). On the Effect of pH, Temperature, and Surfactant Structure on Bovine Serum Albumin–Cationic/Anionic/Nonionic Surfactants Interactions in Cacodylate Buffer–Fluorescence Quenching Studies Supported by UV Spectrophotometry and CD Spectroscopy. Int. J. Mol. Sci..

[44] Li R., Wu Z., Wangb Y., Ding L., Wang Y. (2016). Role of pH-induced structural change in protein aggregation in foam fractionation of bovine serum albumin. Proc. Biotechnol. Rep..

[45] Roberts S.A., Kellaway I.W., Taylor K.M., Warburton B., Peters K. (2005). Combined surface pressure-interfacial shear rheology study of the effect of pH on the adsorption of proteins at the air-water interface. Langmuir.

